# A case report: Marfan syndrome with X trisomy and FBN1 and SDHB mutations

**DOI:** 10.1186/s12920-023-01551-6

**Published:** 2023-05-27

**Authors:** Jiansheng Lin, Yanyu Lin, Gaoxiong Wang

**Affiliations:** 1 Department of Laboratory Medicine, Quanzhou Women’s and Children’s Hospital, Quanzhou, People’s Republic of China; 2 Department of Ophthalmology, Quanzhou Women’s and Children’s Hospital, Quanzhou, People’s Republic of China; 3 Quanzhou Women’s and Children’s Hospital, Quanzhou, People’s Republic of China

**Keywords:** Marfan syndrome, X trisomy, FBN1, SDHB, Case report

## Abstract

**Background:**

Marfan syndrome (MFS) is a rare autosomal dominant connective tissue disorder affecting the cardiovascular, skeletal, and ophthalmic systems. This report aimed to describe a novel genetic background and treatment prognosis of MFS.

**Case presentation:**

A proband was initially diagnosed with bilateral pathologic myopia and suspected MFS. We performed whole exome sequencing and found a pathogenic nonsense *FBN1* mutation in the proband, which confirmed the diagnosis of MFS. Notably, we identified a second pathogenic nonsense mutation in *SDHB*, which increased the risk of tumours. In addition, the proband karyotype was X trisomy, which may cause X trisomy syndrome. At the 6-month follow-up after posterior scleral reinforcement surgery, the proband's visual acuity improved significantly; however, myopia was still progressing.

**Conclusions:**

We report a rare case of MFS with a X trisomy genotype, a mutation in *FBN1* and a mutation in *SDHB* for the first time, and our findings could be helpful for the clinical diagnosis and treatment of this disease.

## Background

MFS is a rare autosomal dominant connective tissue disease with an incidence of approximately 1 in 5000, and its clinical symptoms mainly involve the cardiovascular, skeletal, and ophthalmic systems [1]. In most cases, MFS is caused by mutations in *FBN1*. *FBN1* encodes the major component of extracellular microfibrils, which interfere with local transforming growth factor β signalling and disrupt tissue integrity [2]. Moreover, gene mutation detection is suitable for early diagnosis if clinical symptoms do not meet diagnostic criteria [3]. Rare genetic events and novel mutations that are disease associated (particularly for monogenic diseases) and potential disease-causing mutations due to incomplete penetrance can be detected by whole exome sequencing (WES) [4]. In this study, we aimed to describe the specific genetic background and treatment prognosis of MFS.

## Case presentation

A 4-year-old girl with normal weight (18 kg) who was slightly taller than girls of the same age (118 cm) complained at the initial consultation. Poor binocular vision was found during physical examination 1 year prior, with no other abnormalities detected. The patient's myopia gradually progressed, and she came to our hospital for treatment. The preliminary diagnosis was bilateral pathologic myopia and suspected MFS. Past history revealed no hypertension, diabetes, other eye diseases, infectious diseases, trauma, or surgery. Birth history: the patient was born via a full-term natural delivery at a birth weight of 2.8 kg, with no history of asphyxia after birth. The G1P1 mother was healthy during pregnancy and denied any history of medication use and X-ray exposure.

**Fig. 1:**
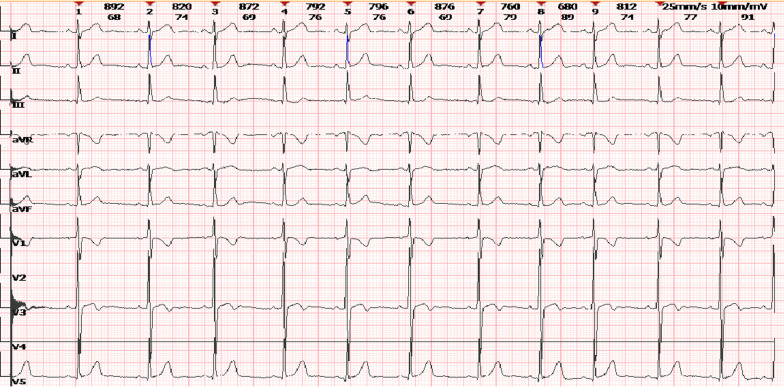
Detection record of routine electrocardiogram

**Fig. 2:**
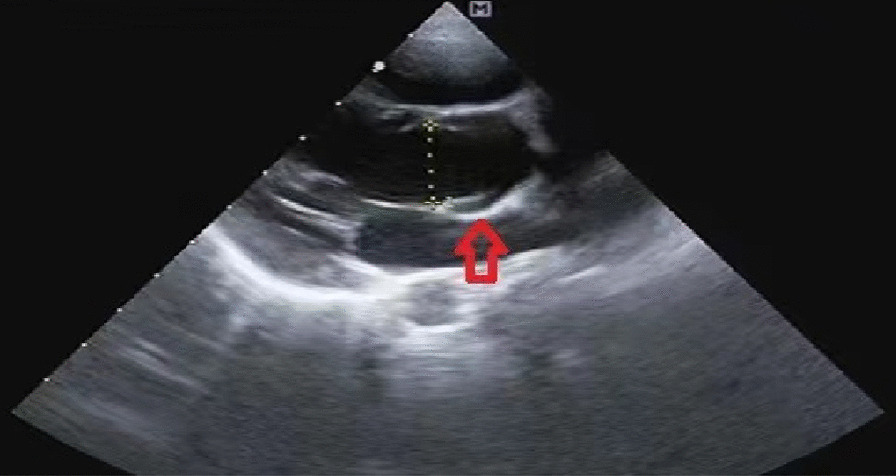
Echocardiographic image of aortic root. *Note*: the red arrow indicates aortic sinus of Valsalva

Preoperative physical examination: The patient exhibited stable vital signs. The visual acuity with glasses was 0.25 (mydriatic refraction, − 7.00/− 1.50 * 15 → 0.2) for oculus dexter (OD) and 0.25 (mydriatic refraction, − 4.00/− 0.75 * 160 → 0.25) for oculus sinister (OS). The intraocular pressure was 11 mmHg for OD and 13 mmHg for OS. The axial length was 25.49 mm in the right eye and 23.95 mm in the left eye. Other ophthalmic examinations, including lentis, were normal. Auxiliary examination: Routine electrocardiogram showed sinus bradycardia with arrhythmia, as shown in Fig. 1. Echocardiography revealed that because the diameter of the aortic Valsalva sinus was 2.18 cm, which is greater than the upper limit of the reference value of 2.0 cm, the aortic sinus was slightly dilated, as shown in Fig. 2. The aortic root Z value of 2.078 was slightly greater than the upper limit of the reference value of 2, which suggested aortic dilatation, and left ventricular systolic function was normal. Routine haematuria, coagulation, biochemical, and chest X-ray evaluations revealed no abnormalities.

After hospitalization, we performed posterior scleral reinforcement surgery, which was successful. Condition at discharge: visual acuity, OD: 0.4 and OS: 0.5; intraocular pressure, 15 mmHg OD and 15 mmHg OS; mild oedema of the eyelids; and hyperaemia and oedema of the conjunctiva (+). Other eye examinations were normal. To confirm the diagnosis, peripheral blood was drawn from the proband for WES.

At follow-up after 6 months, the proband’s visual acuity with glasses was 0.5 (mydriatic refraction, − 8.50/− 2.50 * 15) for OD and 0.6 (mydriatic refraction, − 4.50/− 1.00 * 155) for OS. The axial length was 25.98 mm in the right eye and 24.28 mm in the left eye. Her parents were basically satisfied with the results of the operation.

## Genetic analysis

WES was performed using the Illumina NovaSeq PE150 platform. Sequencing quality control parameters were as follows: the coverage of the target region was > 99.69%, and the average sequencing depth of the target region was 144.14×. We sequenced the entire human exome, covering > 20,000 genes and > 85% of human genetic diseases. Read-depth-based copy number variation (CNV) was analysed by CNVkit software (https://cnvkit.readthedocs.io/en/stable/). A copy number of 3 was considered a triploid duplicate.

**Fig. 3:**
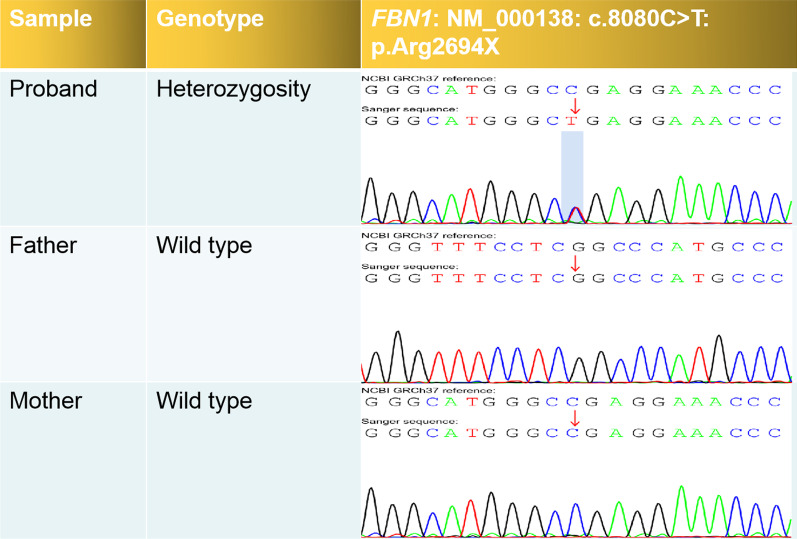
Validation of FBN1 gene mutation

The proband phenotype (high myopia, sinus bradycardia with arrhythmia, slightly dilated aortic sinus, slender lower extremities, and loose ligaments of the limbs) was consistent with MFS caused by an *FBN1* mutation. In particular, we identified a nonsense mutation in exon 65 of *FBN1* caused by the substitution of cytosine (C) to thymine (T) at position 8080 of the cDNA. This mutation resulted in the substitution of arginine (Arg) to a stop codon at position 2694, as shown in Table 1. As demonstrated in Fig. 3, Sanger sequencing was used to confirm the *FBN1* mutation. Moreover, pedigree analysis revealed that this heterozygous variant in the proband was a de novo variant. According to the 2015 American College of Medical Genetics and Genomics (ACMG) guidelines, the pathogenic evidence of this variant was “PVS1 + PS2 + PS4 + PM2_Supporting;” thus, we classified it as pathogenic. Additionally, the heterozygous *FBN1* variant in the proband conformed to the autosomal dominant (AD) inheritance pattern of MFS. The nonsense *FBN1* mutation confirmed the diagnosis of MFS.

**Table 1:** Information on gene variant sites

Gene	Chromosomal location	rs number	Variant site	Types of mutation	Proband	Father	Mother	ACMG Pathogenicity RATING	Related diseases (OMIM)	Genetic pattern
*FBN1*	chr15: 48704912	rs 200309328	NM_000138: exon65: c.8080C > T: p.Arg2694*	Nonsense	Heterozygous	Wild type	Wild type	Pathogenic	MFS (154,700)	AD
*SDHB*	chr1: 17371320	rs 74315370	NM_003000: exon2: c.136C > T: p.Arg46*	Nonsense	Heterozygous	Heterozygous	Wild type	Pathogenic	Paraganglioma type 4 (115,310)	AD

*ACMG* American College of Medical Genetics and Genomics, *AD* autosomal dominant

* indicates that an amino acid at a certain position becomes a stop codon

**Fig. 4:**
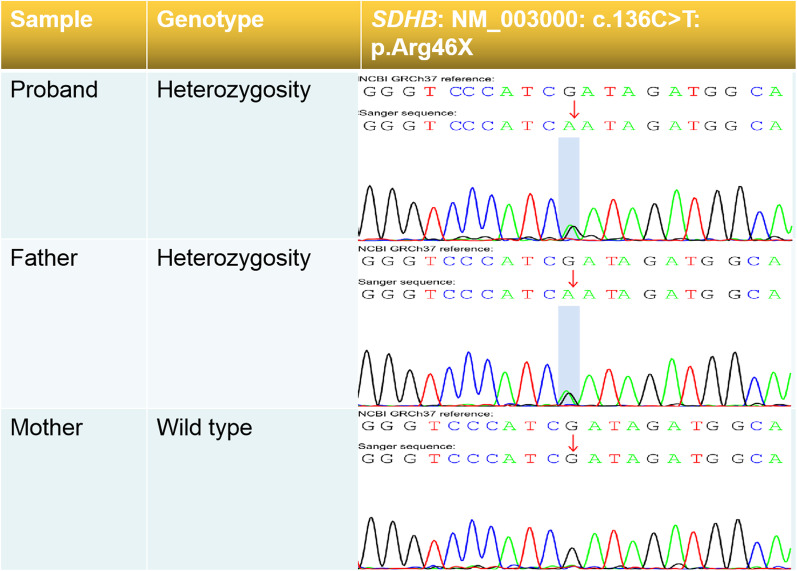
Validation of SDHB gene mutation

**Fig. 5:**
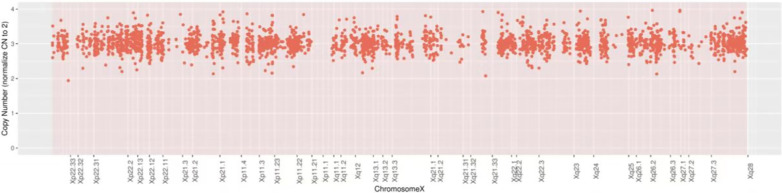
Detection results for 3 chromosome X. *Note*: the abscissa represents the schematic diagram of the position of the chromosome X. Yellow dots represent detection sites. The copy number 3 on the vertical axis means that the number of chromosome X is 3

In addition to the *FBN1* mutation, we identified two other genetic alterations. The first was an single nucleotide variation in *SDHB*, which was a secondary finding because the clinical phenotype of the *SDHB* mutation did not correspond to the clinical symptoms of the proband. In particular, we identified a nonsense mutation in exon 2 of *SDHB* caused by the substitution of C to T at position 136 of the cDNA, resulting in the substitution of Arg at position 46 to a stop codon, as shown in Table 1. As demonstrated in Fig. 4, Sanger sequencing was used to confirm the *SDHB* mutation. Pedigree analysis revealed that this heterozygous mutation was inherited from the father and that it conformed to the AD inheritance pattern of paraganglioma (PGL) type 4 and pheochromocytoma (PCC). Therefore, the variant was classified as pathogenic (PVS1 + PS4 + PM2_Supporting), according to the ACMG guidelines. The other genetic alteration we identified was a CNV, particularly a copy number duplication at the Xp22.33-Xq28 region containing 1154 genes, as shown in Table 2 and Fig. 5. This 155 Mb duplication was classified as pathogenic according to the 2019 ACMG criteria for CNVs. The genetic syndrome associated with this region is Superfemale syndrome.

**Table 2:** Details of the repeating region of the break point of the X chromosome

Chromosomal region	CNV type	Fragment size (bp)	Chromosomal location	Contains major genes
Xp22.33-Xq28	Duplication (CNV = 3)	155,072,003	chrX: 60,500 -155,132,503	*ABCB7*, *ABCD1*, *ACE2*, *ACOT9*, *ACSL4*,*ACTRT1*,*ADGRG2*, *ADGRG4*,*AFF2*,*AGTR2* etc. including 1154 genes

Xp22.33-Xq28 covers the entire X chromosome

## Discussion and conclusions

MFS involves multiple systems [5], and vision loss and skeletal system abnormalities are typically detected earlier than cardiovascular abnormalities [4]. These features are consistent with this case, as severe vision loss was the first symptom we detected in this proband. A previous study revealed that posterior scleral reinforcement surgery in patients with MFS and retinoschisis results in retinal reattachment [6]. Notably, the proband's visual acuity improved significantly after surgery; in particular, it increased from 0.25 to 0.5 OD and from 0.25 to 0.6 OS. However, myopia progressed from − 7.00 to − 8.50 OD (mydriatic refraction) and from − 4.00 to − 4.50 OS (mydriatic refraction). One possible explanation is that surgery promoted retinal angiogenesis but could not inhibit axial growth, as the axial length increased from 25.49 to 25.98 mm in the right eye and from 23.95 to 24.28 mm in the left eye.

According to the Online Mendelian Inheritance in Man (OMIM) database, *FBN1* mutations (OMIM * 134,797) can cause eight diseases, including acromicric dysplasia, MFS, and stiff skin syndrome. We diagnosed MFS based on the proband phenotype, which was consistent with that of MFS. In addition to being associated with *FBN1*, MFS is also associated with mutations in *TGFBR1* and *TGFBR2*. Through WES technology, more genes that may be related to MFS, such as *TTN* and *POMT1*, can be identified [7], and a small, 0.76 Mb microdeletion of CNV in 15q21.1 leading to haploinsufficiency of the fibrillin 1 (*FBN1*) gene associated with MFS was discovered [8]. In view of the advantages of WES in the diagnosis of MFS, WES was used for the genetic diagnosis of this proband. The heterozygous *FBN1* variant p.R2694* we identified in the proband has a 50% chance of being inherited by the next generation. Since the *FBN1* mutation was a de novo variant, the likelihood of this mutation occurring in the parents’ next child is very small, considering the possibility of germ cell mosaicism in one parent.

*SDHB* mutations (OMIM * 185,470) are related to gastrointestinal stromal tumours, mitochondrial complex II deficiency, PGL, gastric stromal sarcoma, and PCC and are associated with a high rate of malignancy in PGL and PCC [9]. The overall penetrance of *SDHB* mutations is approximately 21% at the age of 50 and 42% at the age of 70 [10]. This is likely why the proband and her father had no symptoms of PGL and PCC. We recommend follow-up of the proband and her father to assess the clinical phenotype and perform relevant examinations, if necessary, with the aim of further clarifying the clinical significance of *SDHB* mutations. X trisomy syndrome was first described by Jacobs et al. [11], According to recent statistics, the incidence of X trisomy syndrome is approximately 11 in 10,000 women, and only approximately 13% of patients are diagnosed [12]. Females with X trisomy syndrome have a relatively mild form of the disease and do not have typical phenotypic features, which could explain why the diagnosis is easily missed [13]. This 4-year-old proband did not show X trisomy syndrome. In contrast to the symptoms reported in the two previous cases of MFS with X trisomy syndrome [14, 15], we found no immune abnormalities or dysmorphia in the proband, likely due to different genetic alterations. The limitation of this study is that trisomy X was not further verified by karyotype analysis.

In conclusion, this proband was diagnosed with MFS with the X trisomy genotype, a mutation in the *FBN1* gene and a mutation in *SDHB*. Our findings could be helpful for the clinical diagnosis and treatment of this disease.

## Data Availability

The datasets generated during the current study are available in the National Center of Biotechnology Information (NCBI), and the accession number SUB12917819 is under review.

## Abbreviations

MFS: Marfan syndrome
WES: Whole exome sequencing
OD: Oculus dexter
OS: Oculus sinister
ACMG: American College of Medical Genetics and Genomics
C: Cytosine
AD: Autosomal dominant
T: Thymine
Arg: Arginine
PGL: Paraganglioma
PCC: Pheochromocytoma
CNV: Copy number variation
